# The Story behind the Science: Shining a light on the path to discovery

**DOI:** 10.1128/mbio.03679-24

**Published:** 2025-01-21

**Authors:** Arturo Casadevall, Lorraine F. Clark

**Affiliations:** 1 Department of Molecular Microbiology and Immunology, Johns Hopkins Bloomberg School of Public Health, Baltimore, Maryland, USA; 2 American Society for Microbiology, Washington, DC, USA

**Keywords:** history, editorial, respiratory syncytial virus

## EDITORIAL

Researchers turn to scientific literature to inform themselves and stay up to date on the newest developments in their field by reading research articles and reviews. They also contribute to the body of work by publishing their own research results or writing expert reviews. It is standard practice for scientific articles, whether they report original research or evaluate progress on the current state of a scientific discipline, to purely focus on the science. For research articles, authors generally start out with relevant background information on a given topic to provide the lay of the land before introducing the purpose and hypothesis of their own work. In subsequent sections, authors describe the materials and methods used in the experiments in meticulous detail and then present and discuss their research results. Review articles usually provide an overview of a specific research topic or field and describe current progress. However, research papers are usually written in a manner divorced from the actual conduct of research, which is often messy and seldom follows a linear trajectory from hypothesis to discovery. This led Peter Medawar to question whether the typical scientific paper was a fraud (1) since it presented an account of discovery that was very different from what actually happened in laboratory research.

What the scientific literature does not capture is what happens behind the scenes, i.e., the backstory of a scientific breakthrough, such as personal anecdotes or the serendipity that often accompanies discoveries. This information is usually known only to the participants in the discovery and is passed down in an oral tradition to collaborators and trainees. However, like all oral histories, it is vulnerable to the passing of time and the participants. To address this gap in the field of microbiology, *mBio* introduces a new article series called “The Story behind the Science” that will feature papers discussing the history of a discovery or of a microbiology subfield and bring to light information generally not available in the literature. We are interested in anecdotes, human stories, unrecorded details, reminiscences, coincidences, serendipity, and any other ingredient that led to a major discovery. All papers in this series will be collated into an ongoing article collection that can be found on the journal’s collection page (https://journals.asm.org/journal/mbio/collections). The Story behind the Science papers are a subset of the journal’s Commentary article type and are 1,000–1,500 words in length.

Recently, the Johns Hopkins Bloomberg School of Public Health was designated by the American Society for Microbiology as a “Milestones in Microbiology” site for its contributions to microbiology and immunology. When the history of the department of Molecular Microbiology and Immunology was researched for the application, it was learned that respiratory syncytial virus, commonly known as RSV, had been discovered in 1957 in the department as part of a collaboration between the laboratories of Drs. Robert Chanock and Bernard Roizman (2). This information was largely unknown to the faculty since the details of the discovery had been forgotten, and the episode reinforced to one of us (A.C.) the need to work harder to preserve history.

With this editorial, we are introducing the first The Story behind the Science contribution that delves into this discovery of respiratory syncytial virus. Authors A. Casadevall, P. Roane, T. Shenk, and B. Roizman take the reader back to the 1950s and set the stage for the RSV story by describing the state of the art in virology at the time (2). They revisit the simultaneous discovery of the virus that would eventually be named RSV at two separate locations, recount details on experimental procedures relevant to this scientific breakthrough, and note how crucial the RSV discovery was for vaccine development. In revisiting that history, we note the critical contribution made by Dr. Philip Roane, who was a technician at the time and was not an author of the 1957 paper. In discussing the isolation with him, he told us about Dr. Chanock’s interest in the sucrose gradients and how he rewarded Dr. Roane with a bottle of whiskey, details that bring the human dimension of the discovery to life and are otherwise missing from the literature. The article is accompanied by photos of Drs. Robert Chanock and Philip Roane as they appeared in the 1950s, allowing readers to see the faces behind the researchers who conducted this seminal work.

Previously, we have argued for the need for more history of science in science (3). In creating this new series for *mBio*, we aim to record more history that can illuminate the process of discovery. We hope our readers will enjoy exploring this unique set of articles and consider proposing a The Story behind the Science submission of their own. We welcome pre-submission inquiries, which can be sent to mbio@asmusa.org for consideration.
